# Zearalenone Induces Endoplasmic Reticulum Stress and Modulates the Expression of Phase I/II Enzymes in Human Liver Cells

**DOI:** 10.3390/toxins12010002

**Published:** 2019-12-18

**Authors:** Jee Eun Yoon, Kwang Yong Lee, Jin Sil Seok, Wei Nee Cheng, Hyuk Cheol Kwon, Chang Hee Jeong, Sung Gu Han

**Affiliations:** 1 Toxicology Laboratory, Department of Food Science and Biotechnology of Animal Resources, Konkuk University, Seoul 05029, Korea; jee1512@gmail.com (J.E.Y.); seokjinsil@gmail.com (J.S.S.); herm_es@hotmail.com (W.N.C.); rnjs1024@konkuk.ac.kr (H.C.K.); hello01@konkuk.ac.kr (C.H.J.); 2 R & D department, Morningbio Co., Ltd., Cheonan 31111, Korea; morningbio@daum.net

**Keywords:** zearalenone, hepatotoxicity, oxidative stress, ER stress, phase I/II metabolism, apoptosis

## Abstract

Zearalenone (ZEN) is a mycotoxin produced by *Fusarium* species; however, its mechanisms of action in human livers have not been fully elucidated. Thus, we investigated the toxic mechanisms of ZEN in human liver cells. HepG2 cells were treated with ZEN (0–40 μg/mL) for up to 24 h. A significant decrease in cell viability was observed after treatment with 20 and 40 μg/mL of ZEN, including a significant increase in apoptosis and reactive oxygen species production. ZEN increased GRP78 and CHOP, and eIF2α phosphorylation, indicating ER stress; elevated transcription of the autophagy-associated genes, beclin1 and LC3, and translation of LC3; and increased phase I metabolism by increasing PXR and CYP3A4. The protein expression level of CYP3A4 was higher with ZEN treatment up to 20 μg/mL, but remained at the control level after treatment with 40 μg/mL ZEN. In phase II metabolism, Nrf2 activation and UGT1A expression were increased with ZEN treatment up to 20 μg/mL. Treating cells with an ER stress inhibitor alleviated ZEN-induced cell death and autophagy, and inhibited the expression of phase I/II enzymes. Overall, high ZEN concentrations can modulate the expression of phase I/II enzymes via ER stress and reduced protein levels in human liver cells.

## 1. Introduction

Zearalenone (ZEN) is a non-steroidal estrogenic mycotoxin from *Fusarium* species [1]. ZEN contamination is mainly observed in crops such as corn, rice, wheat, and barley, and exposure to this mycotoxin can lead to genotoxicity, teratogenicity, immunotoxicity, and reproductive disorders [2,3,4]. Although ZEN has been known to induce liver toxicity in cells and animals, there is a lack of toxicological information regarding its detailed mechanisms in human liver cells [5,6].

The liver plays a vital role in detoxifying foreign substances and excreting metabolites from the body [7]. When xenobiotics enter the body, the liver secretes numerous enzymes to convert the xenobiotics into more hydrophilic and polar metabolites [8]. These cellular events are referred to as phase I/II metabolism [8]. In phase I metabolism, cytochrome P450 (CYP) enzymes can catalyze the oxidation of xenobiotics, including mycotoxins [9]. Among the many CYP enzymes, CYP3A4 has a broad substrate specificity and is abundant in the human liver and the small intestine [10]. The expression level of CYP3A4 is regulated by the nuclear hormone receptor, pregnane X receptor (PXR) [10]. The metabolites produced by CYP enzymes activate transcription factors, such as nuclear factor erythroid-derived 2-related factor 2 (Nrf2), and induce phase II enzymes, such as UDP-glucuronosyltransferase (UGT), which transfer hydrophilic conjugates to metabolites [11]. The conjugated metabolites can be easily eliminated from the body through phase III transport, which involves the pumping of xenobiotics out of cells using efflux transporters [12]. 

During the biotransformation of foreign substances, reactive metabolites generate reactive oxygen species (ROS) in phase I metabolism [13]. ROS is linked to endoplasmic reticulum (ER) stress in cells [14]. The altered redox homeostasis causes the accumulation of misfolded and unfolded proteins in the ER lumen [15]. When ER stress occurs, unfolded protein response is activated to resolve the misfolding and unfolding of proteins [15]. However, when cells cannot overcome ER stress, ER stress-induced cell death is initiated through activated pro-apoptotic pathways [16]. However, to adapt to cellular stress conditions, including ER stress, autophagy can be initiated [17]. Autophagy plays physiological roles in eukaryotic cells, including degradation and recycling of proteins and defective organelles, and these events are mediated by specific molecules, such as microtubule-associated protein 1A/1B light chain 3 (LC3), beclin1, and autophagy-related genes [18]. 

Although the cytotoxicity of ZEN has been examined in mammalian cells (e.g., mouse Sertoli TM4 cells, porcine oocytes, and human neuroblastoma SH-SY5Y cells), its association with phase I/II metabolism has not been investigated in human liver cells. Therefore, the purpose of this study was to investigate the molecular mechanisms of ZEN-induced hepatotoxicity (i.e., oxidative stress, ER stress, apoptosis, and autophagy) and their association with phase I/II metabolism in human liver cells. 

## 2. Results

### 2.1. ZEN-Induced Cytotoxicity in Human Liver Cells 

**Figure 1:**
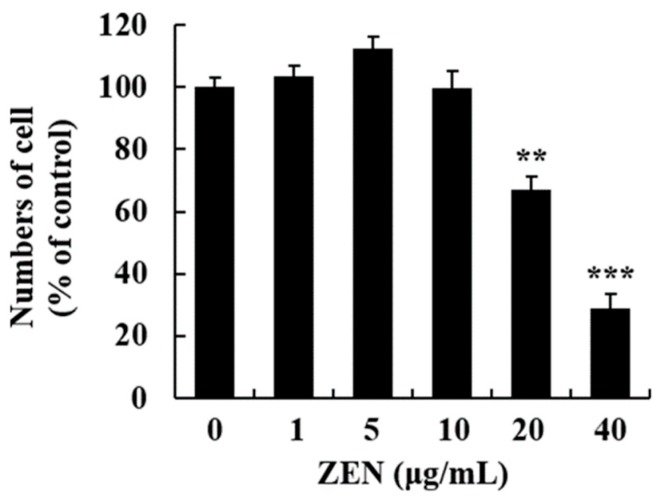
Zearalenone (ZEN) induced cytotoxicity in HepG2 cells. Cells were treated with ZEN (0, 1, 5, 10, 20, and 40 μg/mL) for 24 h and cell viability was measured by the trypan blue dye exclusion test. Data represent mean ± SEM of three independent experiments. * indicates significant difference vs. the control (** *p* < 0.01, and *** *p* < 0.001).

After cells were treated with ZEN (0, 1, 5, 10, 20, and 40 μg/mL) for 24 h, the cytotoxic effect of ZEN on HepG2 cells was examined using the trypan blue dye exclusion test. Based on the results, ZEN significantly decreased cell viability at 20 and 40 μg/mL, compared to the control (Figure 1). However, a marked difference in cell numbers was not observed with ZEN treatment up to 10 μg/mL.

### 2.2. ZEN Increased Apoptosis in Human Liver Cells

**Figure 2:**
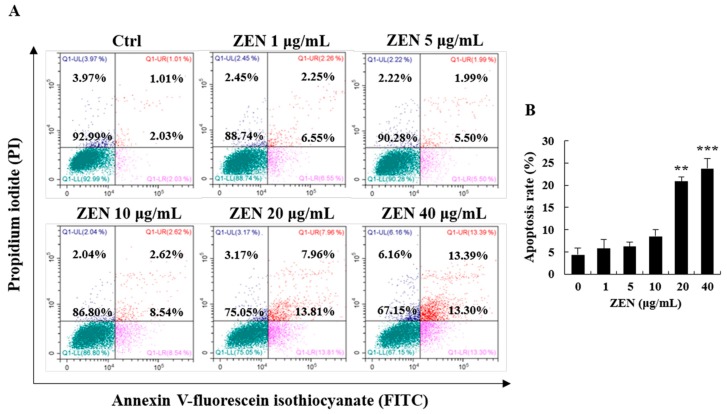
Zearalenone (ZEN) induced apoptosis in HepG2 cells. (**A**) Cells were treated with ZEN (0, 1, 5, 10, 20, and 40 μg/mL) for 24 h and apoptotic/necrotic cells were analyzed using CytoFLEX flow cytometer after Annexin V-FITC/PI double staining. (**B**) The apoptotic rate was calculated using CytExpert software (The parts of Annexin V+/PI- and Annexin V+/PI+ represent early apoptotic cells and late apoptotic/necrotic cells, respectively). The images represent three independent experiments. Data represent mean ± SEM. * indicates significant difference vs. the control (** *p* < 0.01, and *** *p* < 0.001).

The ratio of apoptotic cells was measured using an Annexin V-fluorescein isothiocyanate (FITC)/propidium iodide (PI) double staining assay. The results of flow cytometry analysis revealed that the apoptotic rate was significantly increased at 20 and 40 μg/mL of ZEN (Figure 2A,B). Early apoptotic cells were mainly observed with ZEN treatment at 20 μg/mL, whereas both early apoptotic and late apoptotic cells were observed with 40 μg/mL (Figure 2A,B). There was no marked difference in the rate of apoptotic cells with ZEN treatment up to 10 μg/mL.

### 2.3. ZEN Increased Oxidative Stress, ER Stress, and Autophagy in Human Liver Cells

**Figure 3:**
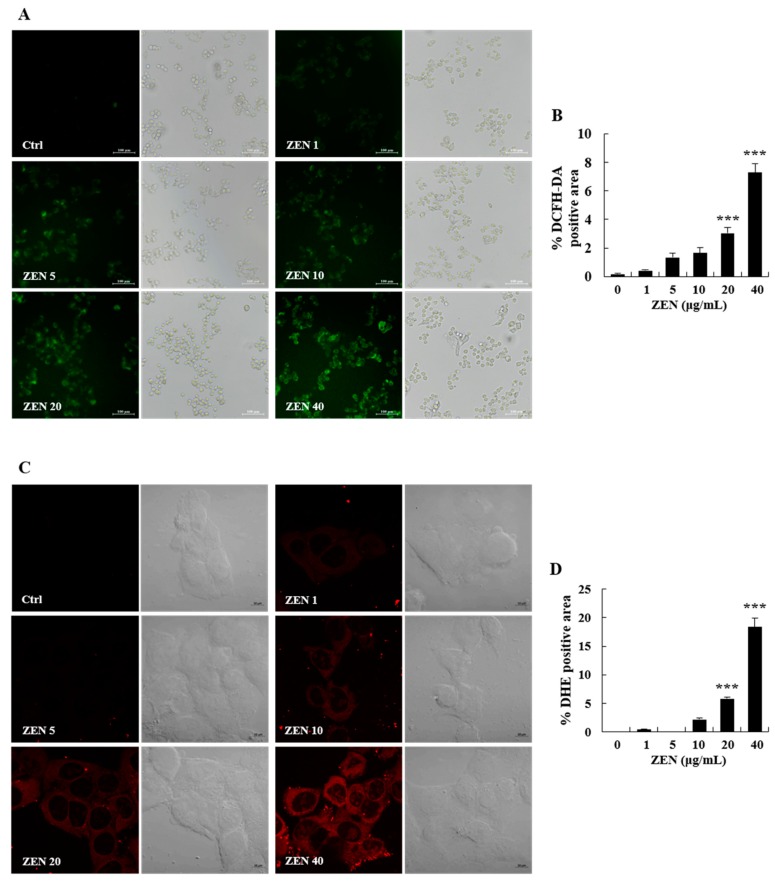
Zearalenone (ZEN) induced oxidative stress in HepG2 cells. Cells treated with ZEN (0, 1, 5, 10, 20, and 40 μg/mL) for 2 h were stained with (**A**) 2′,7′-dichlorofluorescin diacetate (DCFH-DA) and (**C**) dihydroethidium (DHE) to detect intracellular reactive oxygen species (ROS) production. The intensity of green fluorescence (general ROS production) and red fluorescence (superoxide anion production) was assessed by a fluorescence microscope at 200× magnification and a super-resolution confocal laser scanning microscope at 630× magnification, respectively. (**B**) The DCFH-DA positive area (green fluorescence) and (**D**) DHE positive area (red fluorescence) were analyzed using Image J software and graphically presented. The images shown are representatives of three independent experiments. Data represent mean ± SEM. * indicates significant difference vs. the control (*** *p* < 0.001).

**Figure 4:**
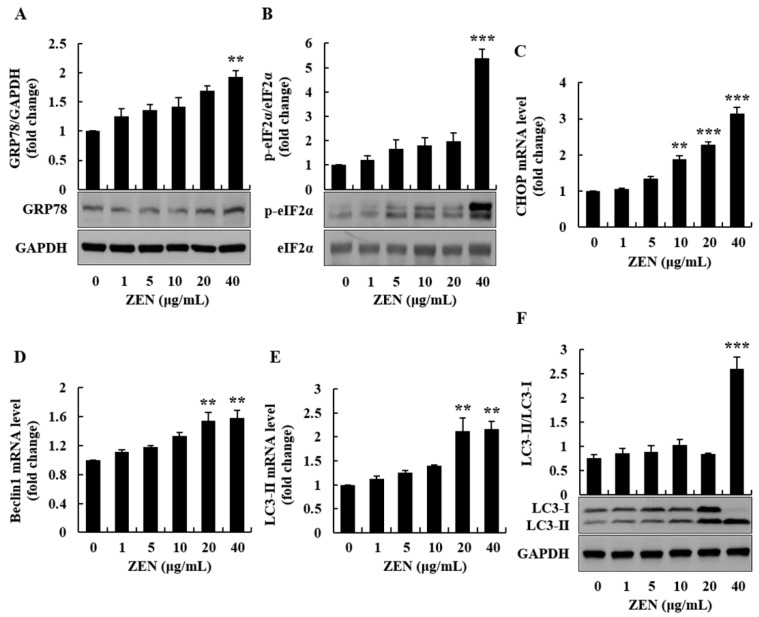
Zearalenone (ZEN) induced endoplasmic reticulum (ER) stress and autophagy in HepG2 cells. mRNA level and protein expression were determined by quantitative real-time polymerase chain reaction (qRT-PCR) and Western blot analysis, respectively. (**A**) The protein expression level of GRP78 was measured in cells treated with ZEN for 1 h. (**B**) The phosphorylation of eIF2α was evaluated in cells treated with ZEN for 2 h. (**C**) CHOP mRNA levels, (**D**) Beclin1 mRNA levels, and (**E**) LC3-II mRNA levels were determined in cells treated with ZEN for 4 h. (**F**) The protein expression level of LC3-II/LC3-I was examined in cells treated with ZEN for 24 h. Data represent mean ± SEM of three independent experiments. * indicates significant difference vs. the control (** *p* < 0.01, and *** *p* < 0.001).

To investigate the underlying mechanisms of ZEN-induced toxicity, oxidative stress was measured using the 2′,7′-dichlorofluorescin diacetate (DCFH-DA) and dihydroethidium (DHE) staining techniques. After 2 h of incubation with ZEN, particularly at 20 and 40 μg/mL, cellular ROS (e.g., superoxide anion) production were increased in a dose-dependent manner (Figure 3A–D). The protein expression level of glucose-related protein 78 (GRP78), a marker of ER stress, was also increased at 40 μg/mL of ZEN (Figure 4A). Furthermore, the phosphorylation of eukaryotic initiation factor 2α (eIF2α), an ER stress-related regulator of the initiation of protein translation, was observed after cells were treated with ZEN. ZEN (40 μg/mL) significantly increased the phosphorylation of eIF2α by approximately 5-fold relative to the control (Figure 4B). These results suggest that a high concentration of ZEN could inhibit protein synthesis through ER stress. 

As ZEN was found to cause ER stress in HepG2 cells, which was demonstrated by the increase in eIF2α phosphorylation, we analyzed the ER stress-related cell signaling molecules linked to apoptosis and autophagy. The mRNA level of human C/EBP homologous protein (CHOP), which is implicated in apoptosis in response to severe ER stress, was measured by quantitative real-time polymerase chain reaction (qRT-PCR). Based on our findings, the level of CHOP mRNA was significantly increased in ZEN-treated cells, particularly at 10, 20, and 40 μg/mL (Figure 4C). The mRNA levels of beclin1 and LC3-II, the autophagy markers involved in the biogenesis of autophagosome, were markedly increased with 20 and 40 μg/mL of ZEN, compared to control (Figure 4D,E). Furthermore, the protein expression level of LC3 was examined by Western blot analysis. As shown in Figure 4F, the LC3-II to LC3-I ratio was significantly increased at 40 μg/mL of ZEN. Such findings indicate that ZEN can induce ER stress, ER stress-induced apoptosis, and autophagy at higher concentrations of ZEN (20 and 40 μg/mL) in human liver cells.

### 2.4. ZEN Induced Phase I/II Metabolism and ER Stress Inhibited Phase I/II Metabolism

**Figure 5:**
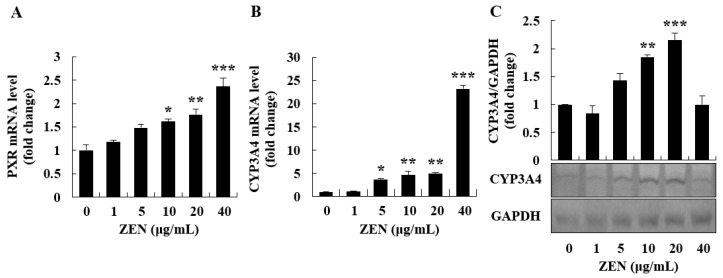
Zearalenone (ZEN) induced the expression of PXR and CYP3A4, and ZEN-induced ER stress inhibited the protein expression of CYP3A4. mRNA level and protein expression were determined by quantitative real-time PCR (qRT-PCR) and Western blot analysis, respectively. (**A**) PXR mRNA levels were determined in cells treated with ZEN for 4 h. (**B**) CYP3A4 mRNA levels were determined in cells treated with ZEN for 8 h. (**C**) The protein expression level of CYP3A4 was examined in cells treated with ZEN for 24 h. Data represent mean ± SEM of three independent experiments. * indicates significant difference vs. the control (* *p* < 0.05, ** *p* < 0.01, and *** *p* < 0.001).

As the induction of xenobiotic defense mechanism may be linked to ER stress, we investigated the cellular responses caused by phase I/II metabolism. The mRNA expression of PXR and CYP3A4, and the protein expression of CYP3A4 were evaluated to identify the effects of ZEN on phase I metabolism. As shown in Figure 5A,B, the mRNA expression of PXR and CYP3A4 were increased in a dose-dependent manner. When treated with 40 μg/mL of ZEN, CYP3A4 mRNA expression level was approximately 20-fold higher than when treated with the control. However, through Western blot analysis, the protein expression level of CYP3A4 was increased at 10 and 20 μg/mL of ZEN, but remained at the control level at 40 μg/mL (Figure 5C). These data suggest that the observed ER stress (Figure 4A–C) may result in the inhibition of CYP3A4 protein synthesis at 40 μg/mL of ZEN. This inability of the CYP3A4 mRNA to be translated to protein may result in a decrease in phase I metabolism when a high concentration of ZEN exists in the liver cells over time. 

**Figure 6:**
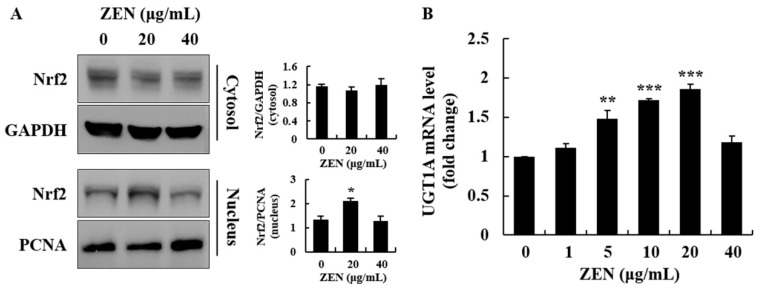
Zearalenone (ZEN) induced Nrf2 activation and UGT1A expression. The mRNA level and protein expression of signaling mediators were determined by quantitative real-time PCR (qRT-PCR) and Western blot analysis, respectively. (**A**) Nrf2 nuclear translocation was measured in cells treated with ZEN (20 and 40 μg/mL) for 6 h by nuclear fractionation and Western blotting. (**B**) UGT1A mRNA levels were determined in cells treated with ZEN for 16 h. Data represent mean ± SEM of three independent experiments. * indicates significant difference vs. the control (* *p* < 0.05, ** *p* < 0.01, and *** *p* < 0.001).

In addition to phase I biotransformation, phase II enzymes are known to metabolize phase I metabolites. Thus, phase II biotransformation-related genes, such as UGT1A, and the activation of the transcription factor, Nrf2, were measured in cells. Translocation of Nrf2 from the cytosol to the nucleus was increased in cells treated with 20 μg/mL of ZEN (Figure 6A). However, the nuclear translocation of Nrf2 returned to control levels at 40 μg/mL of ZEN. Similarly, ZEN increased the mRNA expression of UGT1A up to 20 μg/mL; however, there was no significant increase in UGT1A at 40 μg/mL (Figure 6B). Overall, our data demonstrate that the expression level of the phase I/II enzymes was induced up to 20 μg/mL of ZEN but was inhibited at higher concentrations, such as 40 μg/mL of ZEN, in HepG2 cells.

### 2.5. 4-Phenylbutyric Acid Alleviated ZEN-Induced ER Stress and Autophagy, and Inhibited Phase I/II Metabolism

**Figure 7:**
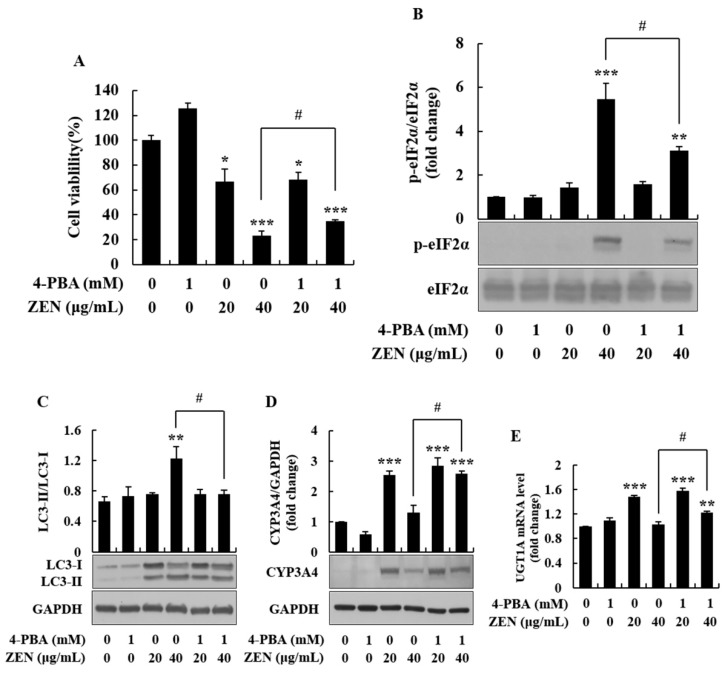
4-phenylbutyric acid (4-PBA) alleviated zearalenone (ZEN)-induced ER stress, autophagy, and downregulation of CYP3A4 and UGT1A. Before treatment with ZEN (20 and 40 μg/mL), cells were pretreated with 1 mM 4-PBA for 6 h. mRNA and protein expression were determined by quantitative real-time PCR (qRT-PCR) and Western blot analysis, respectively. (**A**) Cells were treated with ZEN for 24 h after pretreatment with 4-PBA and cell viability was measured by the trypan blue dye exclusion test. (**B**) Phosphorylation of eIF2α was evaluated in cells treated with ZEN for 2 h. The protein expression of (**C**) LC3-II/LC3-I and (**D**) CYP3A4 was determined in cells treated with ZEN for 24 h. (**E**) UGT1A mRNA levels were determined in cells treated with ZEN for 16 h. Data represent mean ± SEM of three independent experiments. * indicates significant difference vs. the control (* *p* < 0.05, ** *p* < 0.01, and *** *p* < 0.001). # indicates significant difference between groups (*p* < 0.05).

To further confirm the role of ER stress in the hepatotoxicity of ZEN, HepG2 cells were treated with the ER stress inhibitor, 4-phenylbutyric acid (4-PBA), prior to ZEN treatment. At a concentration of 1 mM, 4-PBA displayed no significant cytotoxicity (Figure 7A). ZEN-induced phosphorylation of eIF2α and the increase in the LC3-II/LC3-I ratio were significantly attenuated in the presence of 4-PBA, compared to 40 μg/mL of ZEN alone (Figure 7B,C). Furthermore, in the same cell treatment condition, the expression of CYP3A4 and UGT1A was recovered by the ER stress inhibitor (Figure 7D,E). These findings suggest that ZEN-induced autophagy and the inhibition of protein synthesis (CYP3A4 and UGT1A) may be, at least in part, due to the ER stress caused by a high concentration of ZEN (40 μg/mL).

## 3. Discussion

Mycotoxins are toxic secondary metabolites biosynthesized by fungi [19]. Contamination of foods and feedstuffs with mycotoxins not only results in economic losses but also severe health problems in humans and farm animals [20]. Consumption of mycotoxin-contaminated products can lead to mycotoxicosis and accompanying acute or chronic symptoms. These products are carcinogenic, hepatotoxic, immunosuppressive, and teratogenic [21]. Among many mycotoxins, ZEN is well known to possess an estrogenic effect; however, liver toxicity and the detailed mechanisms underlying this effect have not been fully understood. Thus, in the current study, hepatotoxicity and its related molecular pathways were investigated using the human liver cell line, HepG2. 

To determine the effects of ZEN on cell viability, we employed the trypan blue dye exclusion test. Based on the results, cell viability was markedly lower in cells treated with 20 and 40 μg/mL of ZEN. To identify the cellular mechanism of ZEN-induced cell death, apoptosis was measured. The apoptosis level was markedly increased at 20 and 40 μg/mL, which indicates that early apoptosis may be a major mechanism in cells treated with a low dose (20 μg/mL) of ZEN. However, both apoptosis and necrosis occurred when a high dose of ZEN (40 μg/mL) was administered. These results align with those of previous findings wherein the high dose of ZEN predominantly caused late apoptosis and necrosis compared to the low dose in mouse RAW 264.7 macrophages [22,23]. As oxidative stress is frequently involved in cytotoxicity and cell death, the level of ROS was measured using the DCFH-DA and DHE staining methods. As expected, ZEN induced a significant increase in ROS production (e.g., superoxide anion) at 20 and 40 μg/mL. These data suggest that cellular oxidative stress plays a critical role in the observed apoptosis. In previous studies, similar results were observed in HepG2 and porcine IPEC-J2 cells as ZEN generated ROS, reduced antioxidant enzyme activities, and induced cell death in vitro [24,25,26]. 

Cellular oxidative stress is known to induce ER stress [27]. Because ROS production was highly increased in ZEN-induced cells, particularly at 20 and 40 μg/mL, the ER stress-related markers, GRP78 and phosphorylated eIF2α, were measured via Western blot analysis. ZEN increased the expression of GRP78 and the phosphorylation of eIF2α at 5, 10, 20, and 40 μg/mL. In particular, a high concentration of ZEN (40 μg/mL) significantly increased the expression of GRP78 and phosphorylated eIF2α. GRP78 is a master regulator of unfolded protein response that interacts with three ER stress sensors, including protein kinase RNA-like endoplasmic reticulum kinase (PERK) [28]. Upon ER stress, GRP78 is released from the sensors, resulting in eIF2α phosphorylation through PERK, followed by the global inhibition of protein synthesis [29]. Previously, phosphorylation of eIF2α and inhibition of protein synthesis were found to increase the number of apoptotic cells [30]. A past study also reported that excessive superoxide generation at the ER led to ER stress and apoptosis [31]. In fact, ZEN increased ROS level in lymphocytes that led to excessive oxidative modification of proteins, ER stress and apoptosis [32]. Therefore, our findings regarding ZEN-induced apoptotic cells may be due to ER stress. Previously, ER stress was implicated in apoptosis through CHOP, a downstream signaling molecule of eIF2α [33]. Transcriptional activation of CHOP plays an important role in ER stress-induced apoptosis in cells [34]. Furthermore, a prior study reported similar data in human colon HCT116 cells and human kidney HEK293 cells [35]. In this cell culture study, ZEN induced ER stress and ER stress-mediated cell death through the CHOP pathway. Overall, our findings demonstrate that ER stress, CHOP expression, and apoptosis are important adverse cellular signaling pathways in ZEN-exposed human liver cells.

Under stress conditions, the ER responds to stimuli through translational attenuation and upregulation of protein folding capacity [15]. ER stress can also trigger autophagy via the PERK/eIF2α pathway [36]. Autophagy is a catabolic system characterized by sequestration of cytoplasmic organelles and proteins through autophagosomes/lysosomes. In the current study, ZEN increased both autophagy-associated genes, such as beclin1 and LC3-II, at 20 and 40 μg/mL, and the ratio of LC3-II/LC3-I protein at 40 μg/mL. During the biogenesis of autophagosome, beclin 1 forms a complex with the lipid kinase vacuolar protein sorting 34 (VPS34) and VPS15, thereby driving the nucleation of the isolation membrane [37]. Thereafter, membrane elongation and closure are conducted by two ubiquitin-like conjugation systems, including the LC3 conjugation system, where LC3 is converted to its membrane-bound form, LC3-II [38]. The completed autophagosome fuses with lysosome via several molecules, including beclin1 and LC3-II, leading to the generation of autolysosome and degradation of the enwrapped cytosolic components [38]. Transcriptional elevation of beclin1 and LC3 has been reported in cells undergoing autophagy. For example, activation of SIRT1 enhanced autophagy by increasing the mRNA levels of beclin1 and LC3 in cells such as murine osteoblast MC3T3-E1 cells and degenerative human disc nucleus pulposus cells [39,40]. Although autophagy is a cellular defense process, overproduction of autophagic vesicles can cause cell death by catabolizing indispensable portions of cells and activating apoptotic/necrotic programs [41]. Therefore, the increase in cell death, which was observed in cells treated with 20 and 40 μg/mL of ZEN, may be at least in part due to the overproduction of autophagic vesicles. Similar to our data, ZEN could induce autophagy by increasing beclin1, LC3-II, and autophagosomes in mouse and rat testicular cells [42,43]. Other potential mechanisms were reported in a recent publication where ZEN could induce the expression of cytochrome P450 reductase and oxidative stress, resulting in autophagy in porcine intestinal epithelial cells [44]. 

After ingestion, the absorbed ZEN is primarily metabolized by hepatocytes and enterocytes [45]. In human liver microsomes, ZEN activates xenosensor molecules such as PXR, followed by monohydroxylation of ZEN by the CYP superfamily [46]. In this detoxification process, ZEN is converted to more hydrophilic molecules [46]. In a previous study, ZEN activated nuclear receptors (e.g., PXR) and their phase I target genes (e.g., CYP3A4) in human primary hepatocytes [47]. In our study, ZEN increased the mRNA levels of PXR and CYP3A4 in a dose-dependent manner, which suggested the activation of phase I metabolism in HepG2 cells. Biphasic dose-response is the low-dose stimulation and high-dose inhibition of certain endpoints when cells are exposed to xenobiotics [48]. Chemicals such as α-benzene hexachloride produce a biphasic dose-response in rat hepatocarcinogenesis [48]. In the current study, protein expression of CYP3A4 showed a biphasic dose-response. Through Western blot analysis, its protein expression level was found to be significantly downregulated in cells treated with 40 μg/mL of ZEN, whereas its mRNA levels were dose-dependently increased following treatment with up to 40 μg/mL of ZEN. As the mRNA expression level of CYP3A4 was markedly higher at 40 μg/mL, ZEN-induced ER stress was assumed to be associated with the inability to carry out the translational process. These data suggest that the biotransformation process via the CYP enzyme is not actively occurring in cells exposed to a high concentration of ZEN (40 μg/mL). The inability of translation efficiency can be explained by ZEN-induced phosphorylation of eIF2α as observed in the current study. In fact, once cells experience ER stress, eIF2α, a regulatory subunit of eIF2, is phosphorylated by protein kinases, leading to the global inhibition of protein synthesis [49]. In addition to phase I metabolism, phase II metabolism was investigated. This is because phase I/II metabolism is linked to toxic substances in cellular responses. A prior study reported that ZEN can induce phase II metabolism by upregulating the transcription factor, Nrf2, and its target gene, UGT1A, in piglet liver [50]. Our data, however, showed that ZEN increased phase II metabolism-related molecules, such as the activation of Nrf2 and the mRNA level of UGT1A up to 20 μg/mL, but not at 40 μg/mL. These results are similar to those for phase I-associated transcription factor and enzymes, such as PXR and CYP3A4. The ER stress initiated by high concentrations of ZEN (i.e., 40 μg/mL) may be responsible for the decreased expression of phase II metabolism-related molecules (i.e., Nrf2 and UGT1A). Altogether, our findings suggest that a high concentration of ZEN, such as 40 μg/mL, can disrupt the normal detoxification processes (phase I/II metabolism) in human liver cells, potentially via ER stress and a decrease in the expression of the phase I/II enzymes.

4-PBA is a hydrophobic short chain fatty acid that is utilized as an ER stress inhibitor [51]. As a chemical chaperone, 4-PBA interacts with the hydrophobic domains of unfolded proteins and prevents their aggregation to improve protein folding and stability [51]. Previous studies have reported that 4-PBA reduces ER stress and inhibits ER stress-related markers, including p-eIF2α [52,53,54]. In the current study, 4-PBA could ameliorate ZEN-induced cell death and autophagy, and increase the expression of phase I/II enzymes by decreasing ER stress. These data suggest that the hepatotoxicity of ZEN in HepG2 cells is related to ER stress, particularly at a high concentration of ZEN (i.e., 40 μg/mL).

## 4. Conclusions

In conclusion, our findings demonstrated that ZEN can induce cell death (i.e., apoptosis), oxidative stress, ER stress, and autophagy in human liver cells. In addition, cells exposed to high concentrations of ZEN (e.g., 40 μg/mL) were revealed to have a decrease in the protein levels of the phase I/II xenobiotic enzymes, which may impact on the endogenous metabolism of mycotoxins.

## 5. Materials and Methods 

### 5.1. Chemicals and Reagents

Dulbecco’s modified Eagle’s medium (DMEM), fetal bovine serum (FBS), trypsin, and penicillin/streptomycin were obtained from Welgene Inc. (Daegu, Korea). Phosphate buffered saline (PBS) was purchased from Lonza (Walkersville, MD, USA). ZEN was purchased from Cayman Chemical Company (Ann Arbor, MI, USA). Dimethyl sulfoxide (DMSO) and trypan blue solution were purchased from Amresco (Solon, OH, USA). 4-PBA and DCFH-DA were purchased from Sigma (St. Louis, MO, USA). DHE was obtained from Invitrogen (Carlsbad, CA, USA). 

### 5.2. Cell Culture and Treatments

The human liver cell line, HepG2, was maintained in DMEM supplemented with 10% FBS and penicillin/streptomycin in a humidified atmosphere containing 5% CO_2_ at 37 °C. Cells were grown to approximately 70% confluence and synchronized overnight in medium containing 1% FBS before treatment. Cells were treated with ZEN up to 40 μg/mL for different time lengths. To observe the effect of ER stress, cells were cultured with the ER stress inhibitor, 4-PBA, for 6 h; this was followed by media change and treatment with ZEN (20 and 40 μg/mL).

### 5.3. Cell Viability Assay

Cell viability was determined using the trypan blue dye exclusion test. Cells were seeded in 6-well plates and then treated with ZEN (0, 1, 5, 10, 20, and 40 μg/mL) for 24 h. The number of viable cells was manually counted using a hemocytometer (Hausser Scientific, Horsham, PA, USA).

### 5.4. Analysis of Cell Apoptosis

The ratio of cell apoptosis was analyzed using an annexin V-FITC apoptosis detection kit (Koma Biotech, Seoul, Korea) according to the manufacturer’s protocol. Cells were treated with ZEN for 24 h and washed with PBS after the cell medium, including floating cells, was collected. The attached cells were trypsinized, and the collected cells were washed with serum-containing medium and cold PBS. Cells were then resuspended in 500 μL binding buffer, followed by the addition of 1.25 μL annexin V-FITC (final concentration, 0.5 μg/mL) and incubation for 15 min in the dark. After washing with binding buffer, 10 μL PI was added (final concentration, 0.6 μg/mL) and cells were immediately examined using a CytoFLEX flow cytometer (Beckman Coulter, Indianapolis, IN, USA). In each treatment group, 10,000 cells were counted, and the ratio of apoptosis was analyzed using the CytExpert software (Beckman Coulter, Indianapolis, IN, USA). Four types of cell populations were detected in a dot plot of annexin V-FITC/PI staining: live cells (annexin V−/PI−), early apoptotic cells (annexin V+/PI−), late apoptotic or necrotic cells (annexin V+/PI+), and necrotic cells (annexin V−/PI+).

### 5.5. Assessment of ROS Levels

Intracellular ROS production was measured using DCFH-DA as described previously [55]. Briefly, HepG2 cells were grown to confluence in 6-well plates and treated with ZEN for 2 h. Following treatment, cells were incubated with a final concentration of 10 μM DCFH-DA for 30 min. After two rounds of washing with cold PBS, the production of ROS (H_2_O_2_) was visualized using an Olympus IX71 fluorescence microscope and images were digitally captured with an Olympus DP71 camera and DP controller software (Olympus Optical Co., Ltd, Tokyo, Japan). To measure the production of intracellular superoxide anion, HepG2 cells were treated with ZEN for 2 h. The cells were then incubated with a fluorogenic dye, DHE (1 μM, 30 min), followed by cold PBS washes. The cells on coverslips were fixed with 4% formaldehyde and visualized with a super-resolution confocal laser scanning microscope (Carl Zeiss Co., Ltd, Oberkochen, Germany). Quantification of DCFH-DA positive area (green fluorescence) and DHE positive area (red fluorescence) was conducted using Image J software (National Institute of Health, Bethesda, MD, USA) and graphically presented.

### 5.6. Preparation of Cell Lysate, SDS-PAGE, and Western Blot Analysis

Cells were lysed in RIPA buffer containing 50 mM Tris (pH 8.0), 150 mM NaCl, 1% Triton X-100, 0.5% sodium deoxycholate, 0.1% SDS, and a protease inhibitor mixture (2 μg/mL aprotinin, 10 μg/mL leupeptin, 1 mM PMSF, 5 mM EDTA, 1 mM EGTA, 10 mM sodium fluoride, and 1 mM sodium orthovanadate). Cell lysates were collected by scraping the culture dishes and centrifuging the samples at 21,000× *g* for 20 min at 4 °C. The supernatants were collected and the protein concentration was analyzed with a BCA protein assay kit (Sigma-Aldrich, St. Louis, MO, USA). Protein samples were stored at −80 °C until use. For Western blot analysis, protein samples (40 μg/mL) were separated using SDS-PAGE and subsequently transferred onto nitrocellulose membranes. Membranes were then blocked with 3% non-fat milk buffer for 1 h 30 min at room temperature, followed by incubation with primary antibodies overnight at 4 °C. The primary antibodies included GRP78 (ADI-SPA-826, Enzo Life Sciences, Farmingdale, NY, USA; 1:1000), eIF2α (#5324, Cell Signaling Technology, Danvers, MA, USA; 1:2000), phospho-eIF2α (#3398, Cell Signaling Technology; 1:1000), LC3 (NB600-1384, Novus Biologicals, Littleton, CO, USA; 1:10000), glyceraldehyde 3-phosphate dehydrogenase (GAPDH) (#ABS-16, Merck, Darmstadt, Germany; 1:20000), CYP3A4 (BML-CR3340, Enzo Life Sciences; 1:20000), Nrf2 (sc-722, Santa Cruz Biotechnology, Santa Cruz, CA, USA; 1:2000), and proliferating cell nuclear antigen (PCNA; sc-7907, Santa Cruz Biotechnology; 1:5000). After washing, membranes were incubated with secondary antibodies conjugated with horseradish peroxidase for 1 h at room temperature and visualized using ECL detection reagents (Thermo Scientific, Waltham, MA, USA). The density of the bands was analyzed using Image J software and normalized to the housekeeping proteins.

### 5.7. Nuclear Fractionation

Cells were seeded in a 10-cm dish and treated with ZEN for 6 h. Cells were lysed in hypotonic buffer solution (20 mM Tris (pH 7.4), 10 mM NaCl, 3 mM MgCl_2_) containing a protease inhibitor mixture. After the addition of 10% Triton X-100, cell lysates were centrifuged at 650× g for 10 min at 4 °C. Pellets were resuspended in cell extraction buffer (100 mM Tris (pH 7.4), 100 mM NaCl, 1% Triton X-100, 10% glycerol, 0.1% SDS) containing a protease inhibitor mixture. The homogenates were centrifuged at 14,000× *g* for 30 min at 4 °C. Pellets were collected as a cytosolic fraction and the supernatants were collected as the nuclear fraction. Aliquots of cytosolic and nuclear fractions were stored at −80 °C until use.

### 5.8. RNA Extraction, Reverse Transcription, and Quantitative Real-Time Polymerase Chain Reaction (qRT-PCR)

Total RNAs were extracted using TRI reagent according to the manufacturer’s protocol (Life Technologies, Gaithersburg, MD, USA). The concentration and purity of the RNA were measured as described previously [56]. Total RNA (2 μg) was reverse transcribed by reverse transcription-PCR in a final volume of 20 μL using the TOPscript^TM^ RT DryMIX kit (Enzynomics, Daejeon, Korea) according to the manufacturer’s protocol. Incubation was performed at 37 °C for 5 min, 50 °C for 60 min, and 95 °C for 5 min. The synthesized cDNA was stored at −80 °C until use. qRT-PCR was performed on three biological replicates and technical duplicates/triplicates of each cDNA sample with the PikoReal 96 real-time PCR system (Thermo Scientific, Waltham, MA, USA) using the following primers: CHOP: forward primer: 5′-GCG CAT GAA GGA GAA AGA AC-3′, reverse primer: 5′-TCA CCA TTC GGT CAA TCA GA-3′; beclin1: forward primer: 5′-CAT GCT CTG GCC AAT AAG ATG GGT-3′, reverse primer: 5′-CGG CAG CTC CTT AGA TTT GTC TGT-3′; LC3-II: forward primer: 5′-GAG AAG CAG CTT CCT GTT CTG G-3′, reverse primer: 5′-GTG TCC GTT CAC CAA CAG GAA G-3′; PXR: forward primer: 5′-GCT GGA ACC ATG CTG ACT TTG T-3′, reverse primer: 5′-AAG TGA TAG CCA GTG GCC TTG T-3′; CYP3A4: forward primer: 5′-TCT TCA CCG TGA CCC AAA GTA CTG-3′, reverse primer: 5′-AGC AAA CCT CAT GCC AAT GCA G-3′; UGT1A: forward primer: 5′-CCT TGG ACG TGA TTG GTT TCC TCT-3′, reverse primer: 5′-GGG TCT TGG ATT TGT GGG CTT TCT-3′; GAPDH: forward primer: 5′-GAC CCC TTC ATT GAC CTC AAC TAC-3′, reverse primer: 5′-ATG ACA AGC TTC CCG TTC TCA G-3′. Each PCR solution (total volume of 20 μL) consisted of 1 μL of cDNA sample, 0.5 μL each of 10 μM forward and reverse primer, 10 μL of 2× Real-Time PCR Smart mix (SolGent, Daejeon, Korea), and 8 μL of RNase-free water. The cycling conditions included a denaturation step at 95 °C for 15 min, followed by 40 PCR cycles at 95 °C for 20 s and 60 °C for 40 s. A melting curve analysis was performed at the end of each PCR program to exclude nonspecific product formation. Real-time fluorescence measurements were carried out every cycle and the change in the threshold cycle (∆Ct) was calculated. The mRNA levels of each sample were quantified using the 2^−ΔΔCt^ method and normalized using GAPDH gene, which served as the endogenous control. 

### 5.9. Statistical Analysis

All experiments were repeated at least three times. Data are expressed as mean ± standard error of the mean (SEM). Statistical significance was determined with SPSS-PASW statistics software version 18.0 for Windows (SPSS, Chicago, IL, USA) by one-way ANOVA and groups were compared using Tukey’s test. A *p*-value less than 0.05 was considered to indicate statistical significance.
